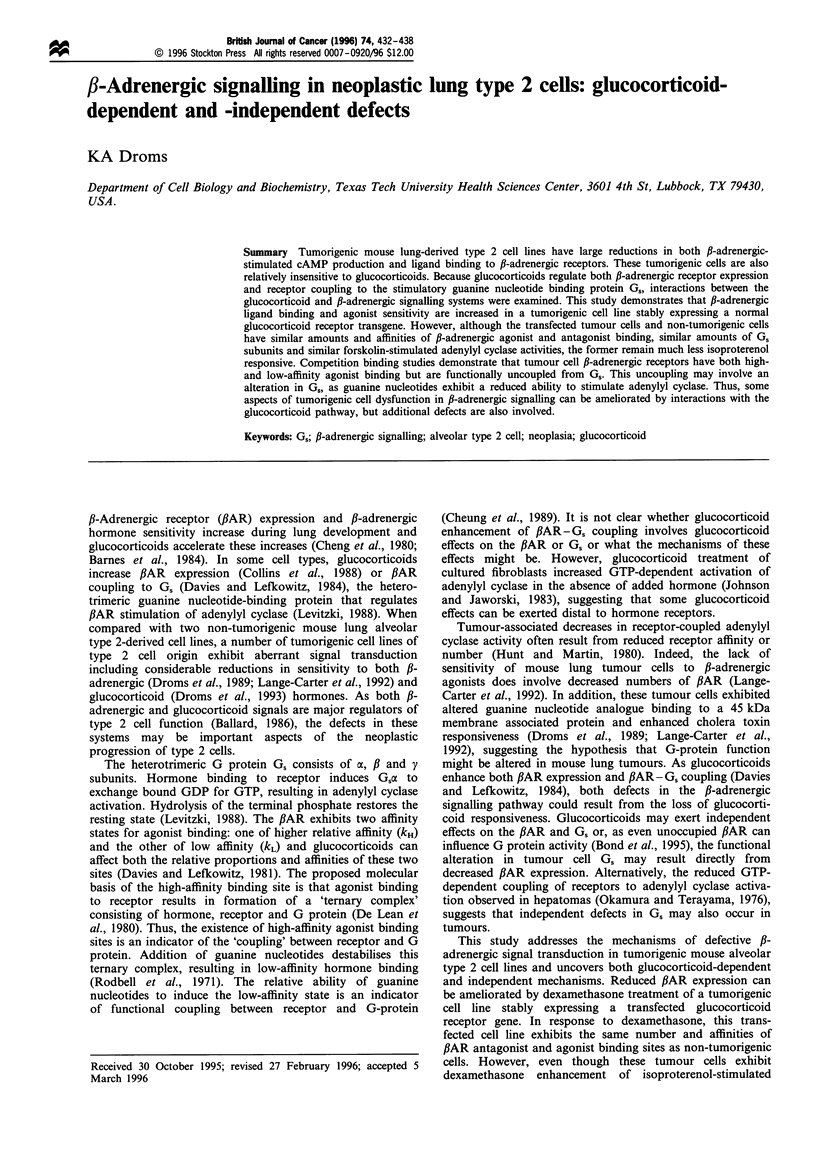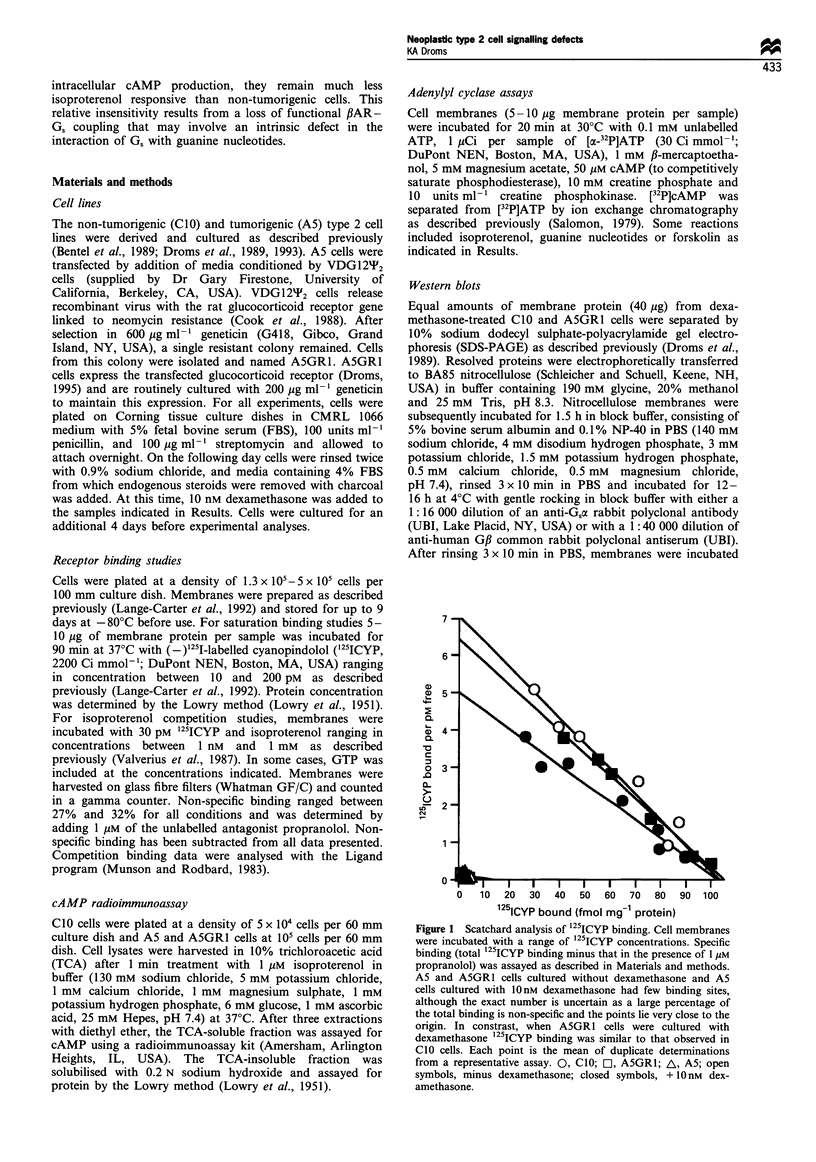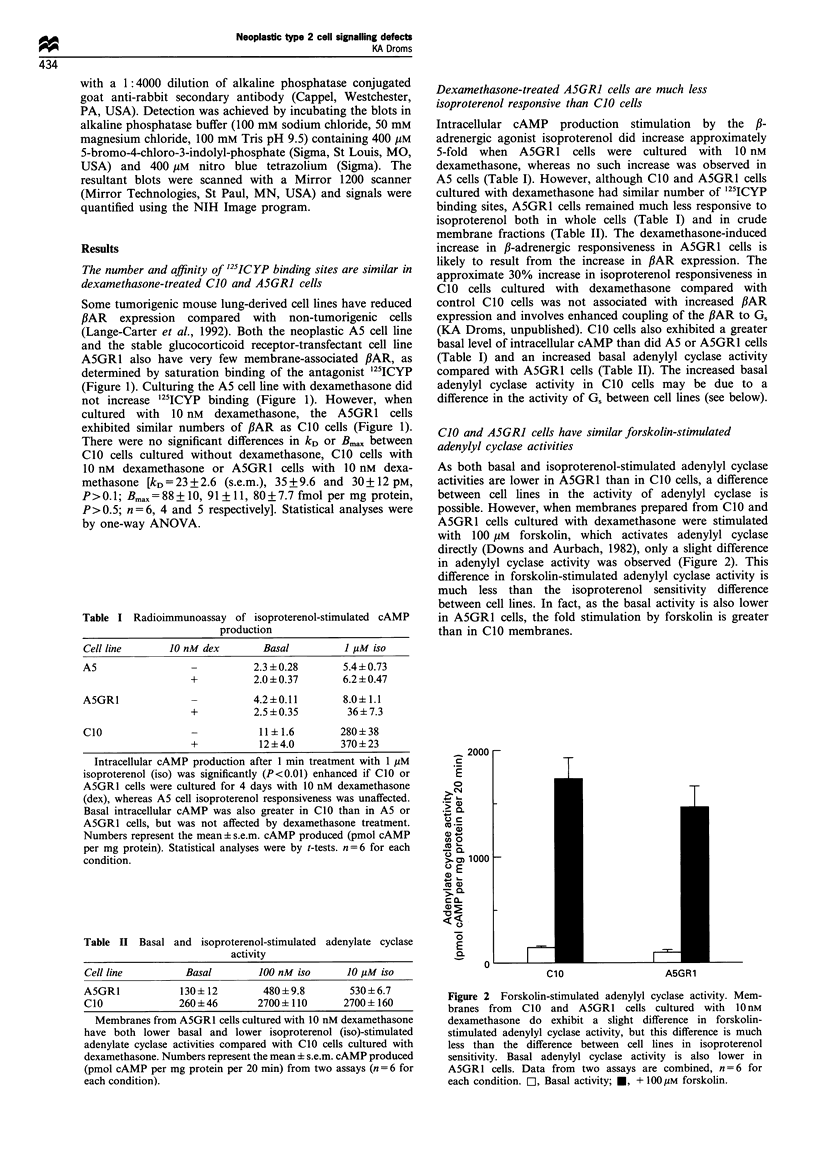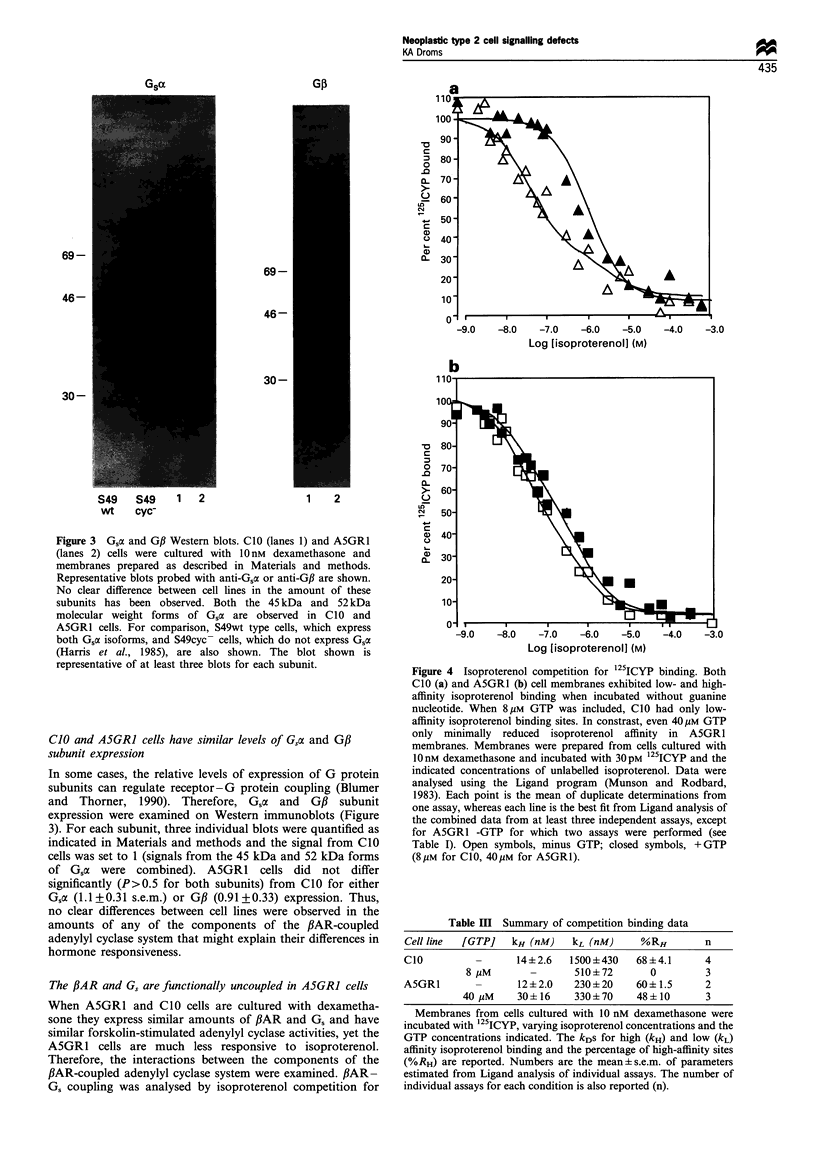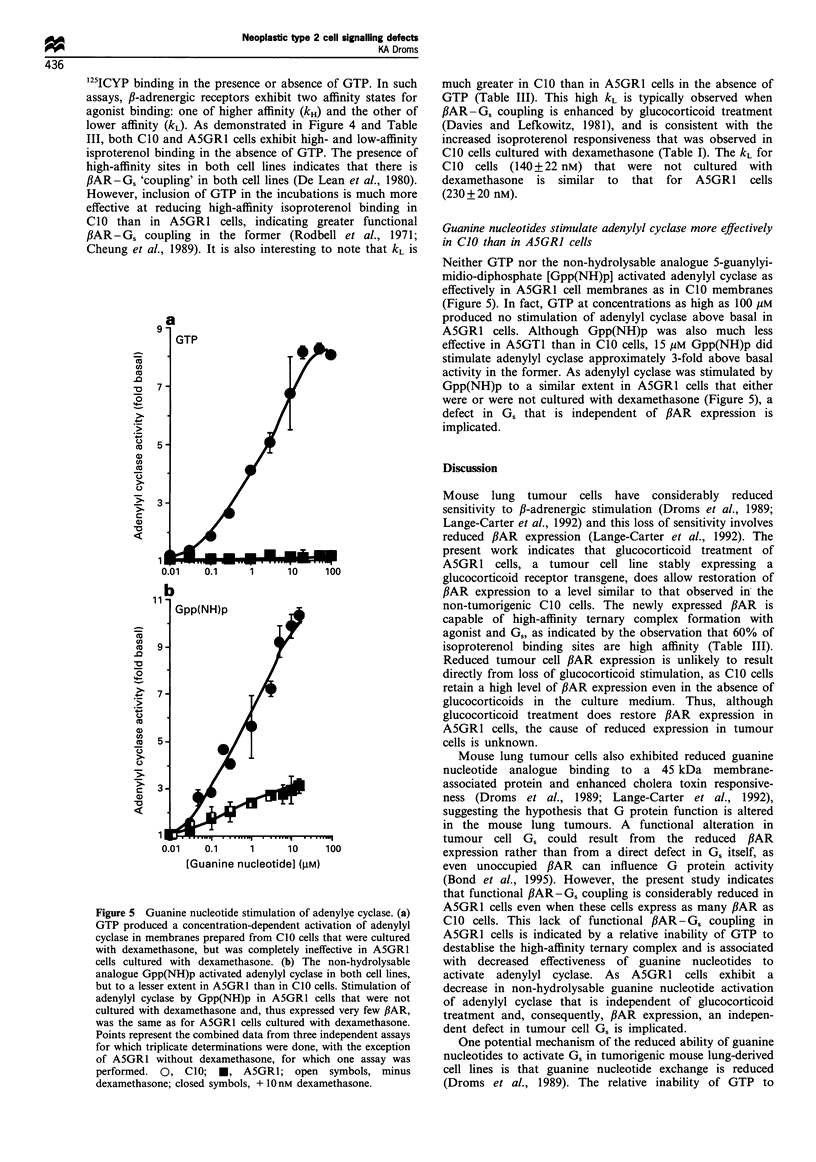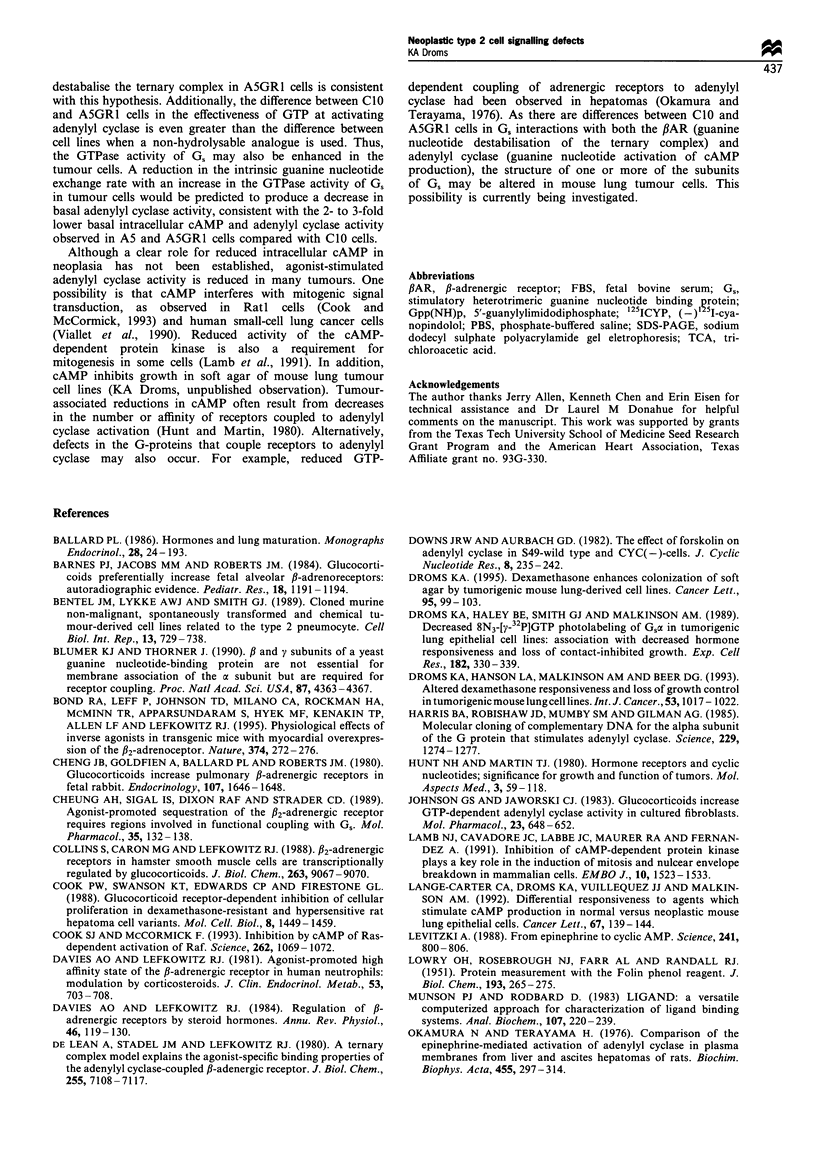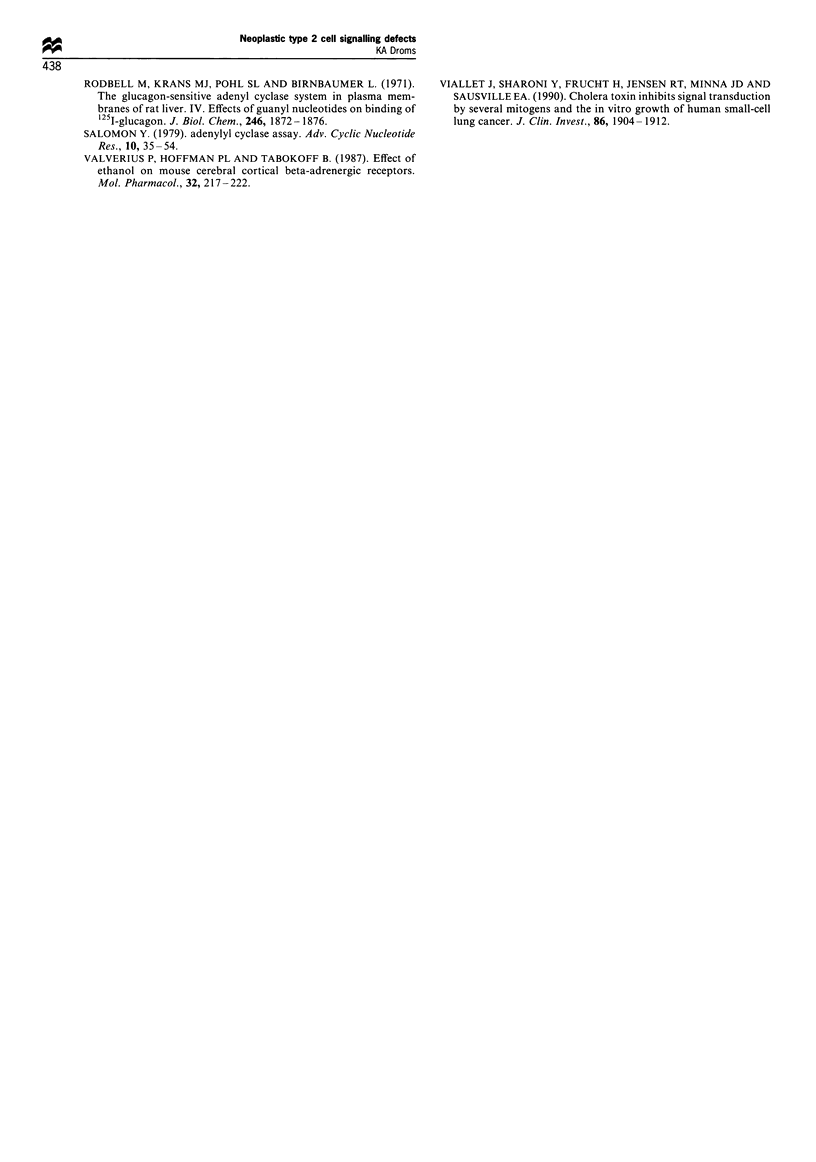# Beta-adrenergic signalling in neoplastic lung type 2 cells: glucocorticoid-dependent and -independent defects.

**DOI:** 10.1038/bjc.1996.377

**Published:** 1996-08

**Authors:** K. A. Droms

**Affiliations:** Department of Cell Biology and Biochemistry, Texas Tech University Health Sciences Center, Lubbock 79430, USA.

## Abstract

**Images:**


British Journal of Cancer (1996) 74, 432–438
© 1996 Stockton Press All rights reserved 0007–0920/96 $12.00

### $\beta$-Adrenergic signalling in neoplastic lung type 2 cells: glucocorticoid-dependent and -independent defects

KA Droms

*Department of Cell Biology and Biochemistry, Texas Tech University Health Sciences Center, 3601 4th St, Lubbock, TX 79430, USA.*

**Summary** Tumorigenic mouse lung-derived type 2 cell lines have large reductions in both $\beta$-adrenergic-stimulated cAMP production and ligand binding to $\beta$-adrenergic receptors. These tumorigenic cells are also relatively insensitive to glucocorticoids. Because glucocorticoids regulate both $\beta$-adrenergic receptor expression and receptor coupling to the stimulatory guanine nucleotide binding protein $G_s$, interactions between the glucocorticoid and $\beta$-adrenergic signalling systems were examined. This study demonstrates that $\beta$-adrenergic ligand binding and agonist sensitivity are increased in a tumorigenic cell line stably expressing a normal glucocorticoid receptor transgene. However, although the transfected tumour cells and non-tumorigenic cells have similar amounts and affinities of $\beta$-adrenergic agonist and antagonist binding, similar amounts of $G_s$ subunits and similar forskolin-stimulated adenylyl cyclase activities, the former remain much less isoproterenol responsive. Competition binding studies demonstrate that tumour cell $\beta$-adrenergic receptors have both high- and low-affinity agonist binding but are functionally uncoupled from $G_s$. This uncoupling may involve an alteration in $G_s$, as guanine nucleotides exhibit a reduced ability to stimulate adenylyl cyclase. Thus, some aspects of tumorigenic cell dysfunction in $\beta$-adrenergic signalling can be ameliorated by interactions with the glucocorticoid pathway, but additional defects are also involved.

**Keywords:** $G_s$; $\beta$-adrenergic signalling; alveolar type 2 cell; neoplasia; glucocorticoid

$\beta$-Adrenergic receptor ($\beta$AR) expression and $\beta$-adrenergic hormone sensitivity increase during lung development and glucocorticoids accelerate these increases (Cheng *et al.*, 1980; Barnes *et al.*, 1984). In some cell types, glucocorticoids increase $\beta$AR expression (Collins *et al.*, 1988) or $\beta$AR coupling to $G_s$ (Davies and Lefkowitz, 1984), the heterotrimeric guanine nucleotide-binding protein that regulates $\beta$AR stimulation of adenylyl cyclase (Levitzki, 1988). When compared with two non-tumorigenic mouse lung alveolar type 2-derived cell lines, a number of tumorigenic cell lines of type 2 cell origin exhibit aberrant signal transduction including considerable reductions in sensitivity to both $\beta$-adrenergic (Droms *et al.*, 1989; Lange-Carter *et al.*, 1992) and glucocorticoid (Droms *et al.*, 1993) hormones. As both $\beta$-adrenergic and glucocorticoid signals are major regulators of type 2 cell function (Ballard, 1986), the defects in these systems may be important aspects of the neoplastic progression of type 2 cells.

The heterotrimeric G protein $G_s$ consists of $\alpha$, $\beta$ and $\gamma$ subunits. Hormone binding to receptor induces $G_s\alpha$ to exchange bound GDP for GTP, resulting in adenylyl cyclase activation. Hydrolysis of the terminal phosphate restores the resting state (Levitzki, 1988). The $\beta$AR exhibits two affinity states for agonist binding: one of higher relative affinity ($k_H$) and the other of low affinity ($k_L$) and glucocorticoids can affect both the relative proportions and affinities of these two sites (Davies and Lefkowitz, 1981). The proposed molecular basis of the high-affinity binding site is that agonist binding to receptor results in formation of a 'ternary complex' consisting of hormone, receptor and G protein (De Lean *et al.*, 1980). Thus, the existence of high-affinity agonist binding sites is an indicator of the 'coupling' between receptor and G protein. Addition of guanine nucleotides destabilises this ternary complex, resulting in low-affinity hormone binding (Rodbell *et al.*, 1971). The relative ability of guanine nucleotides to induce the low-affinity state is an indicator of functional coupling between receptor and G-protein (Cheung *et al.*, 1989). It is not clear whether glucocorticoid enhancement of $\beta$AR–$G_s$ coupling involves glucocorticoid effects on the $\beta$AR or $G_s$ or what the mechanisms of these effects might be. However, glucocorticoid treatment of cultured fibroblasts increased GTP-dependent activation of adenylyl cyclase in the absence of added hormone (Johnson and Jaworski, 1983), suggesting that some glucocorticoid effects can be exerted distal to hormone receptors.

Tumour-associated decreases in receptor-coupled adenylyl cyclase activity often result from reduced receptor affinity or number (Hunt and Martin, 1980). Indeed, the lack of sensitivity of mouse lung tumour cells to $\beta$-adrenergic agonists does involve decreased numbers of $\beta$AR (Lange-Carter *et al.*, 1992). In addition, these tumour cells exhibited altered guanine nucleotide analogue binding to a 45 kDa membrane associated protein and enhanced cholera toxin responsiveness (Droms *et al.*, 1989; Lange-Carter *et al.*, 1992), suggesting the hypothesis that G-protein function might be altered in mouse lung tumours. As glucocorticoids enhance both $\beta$AR expression and $\beta$AR–$G_s$ coupling (Davies and Lefkowitz, 1984), both defects in the $\beta$-adrenergic signalling pathway could result from the loss of glucocorticoid responsiveness. Glucocorticoids may exert independent effects on the $\beta$AR and $G_s$ or, as even unoccupied $\beta$AR can influence G protein activity (Bond *et al.*, 1995), the functional alteration in tumour cell $G_s$ may result directly from decreased $\beta$AR expression. Alternatively, the reduced GTP-dependent coupling of receptors to adenylyl cyclase activation observed in hepatomas (Okamura and Terayama, 1976), suggests that independent defects in $G_s$ may also occur in tumours.

This study addresses the mechanisms of defective $\beta$-adrenergic signal transduction in tumorigenic mouse alveolar type 2 cell lines and uncovers both glucocorticoid-dependent and independent mechanisms. Reduced $\beta$AR expression can be ameliorated by dexamethasone treatment of a tumorigenic cell line stably expressing a transfected glucocorticoid receptor gene. In response to dexamethasone, this transfected cell line exhibits the same number and affinities of $\beta$AR antagonist and agonist binding sites as non-tumorigenic cells. However, even though these tumour cells exhibit dexamethasone enhancement of isoproterenol-stimulated

Received 30 October 1995; revised 27 February 1996; accepted 5 March 1996

intracellular cAMP production, they remain much less isoproterenol responsive than non-tumorigenic cells. This relative insensitivity results from a loss of functional $\beta$AR–$G_s$ coupling that may involve an intrinsic defect in the interaction of $G_s$ with guanine nucleotides.

### Materials and methods

### *Cell lines*

The non-tumorigenic (C10) and tumorigenic (A5) type 2 cell lines were derived and cultured as described previously (Bentel *et al.*, 1989; Droms *et al.*, 1989, 1993). A5 cells were transfected by addition of media conditioned by VDG12$\Psi_2$ cells (supplied by Dr Gary Firestone, University of California, Berkeley, CA, USA). VDG12$\Psi_2$ cells release recombinant virus with the rat glucocorticoid receptor gene linked to neomycin resistance (Cook *et al.*, 1988). After selection in 600 $\mu$g ml$^{-1}$ geneticin (G418, Gibco, Grand Island, NY, USA), a single resistant colony remained. Cells from this colony were isolated and named A5GR1. A5GR1 cells express the transfected glucocorticoid receptor (Droms, 1995) and are routinely cultured with 200 $\mu$g ml$^{-1}$ geneticin to maintain this expression. For all experiments, cells were plated on Corning tissue culture dishes in CMRL 1066 medium with 5% fetal bovine serum (FBS), 100 units ml$^{-1}$ penicillin, and 100 $\mu$g ml$^{-1}$ streptomycin and allowed to attach overnight. On the following day cells were rinsed twice with 0.9% sodium chloride, and media containing 4% FBS from which endogenous steroids were removed with charcoal was added. At this time, 10 nM dexamethasone was added to the samples indicated in Results. Cells were cultured for an additional 4 days before experimental analyses.

### *Receptor binding studies*

Cells were plated at a density of $1.3 \times 10^5 - 5 \times 10^5$ cells per 100 mm culture dish. Membranes were prepared as described previously (Lange-Carter *et al.*, 1992) and stored for up to 9 days at −80°C before use. For saturation binding studies 5–10 $\mu$g of membrane protein per sample was incubated for 90 min at 37°C with (−)$^{125}$I-labelled cyanopindolol ($^{125}$ICYP, 2200 Ci mmol$^{-1}$; DuPont NEN, Boston, MA, USA) ranging in concentration between 10 and 200 pM as described previously (Lange-Carter *et al.*, 1992). Protein concentration was determined by the Lowry method (Lowry *et al.*, 1951). For isoproterenol competition studies, membranes were incubated with 30 pM $^{125}$ICYP and isoproterenol ranging in concentrations between 1 nM and 1 mM as described previously (Valverius *et al.*, 1987). In some cases, GTP was included at the concentrations indicated. Membranes were harvested on glass fibre filters (Whatman GF/C) and counted in a gamma counter. Non-specific binding ranged between 27% and 32% for all conditions and was determined by adding 1 $\mu$M of the unlabelled antagonist propranolol. Non-specific binding has been subtracted from all data presented. Competition binding data were analysed with the Ligand program (Munson and Rodbard, 1983).

### *cAMP radioimmunoassay*

C10 cells were plated at a density of $5 \times 10^4$ cells per 60 mm culture dish and A5 and A5GR1 cells at $10^5$ cells per 60 mm dish. Cell lysates were harvested in 10% trichloroacetic acid (TCA) after 1 min treatment with 1 $\mu$M isoproterenol in buffer (130 mM sodium chloride, 5 mM potassium chloride, 1 mM calcium chloride, 1 mM magnesium sulphate, 1 mM potassium hydrogen phosphate, 6 mM glucose, 1 mM ascorbic acid, 25 mM Hepes, pH 7.4) at 37°C. After three extractions with diethyl ether, the TCA-soluble fraction was assayed for cAMP using a radioimmunoassay kit (Amersham, Arlington Heights, IL, USA). The TCA-insoluble fraction was solubilised with 0.2 N sodium hydroxide and assayed for protein by the Lowry method (Lowry *et al.*, 1951).

### *Adenylyl cyclase assays*

Cell membranes (5–10 $\mu$g membrane protein per sample) were incubated for 20 min at 30°C with 0.1 mM unlabelled ATP, 1 $\mu$Ci per sample of [$\alpha$-$^{32}$P]ATP (30 Ci mmol$^{-1}$; DuPont NEN, Boston, MA, USA), 1 mM $\beta$-mercaptoethanol, 5 mM magnesium acetate, 50 $\mu$M cAMP (to competitively saturate phosphodiesterase), 10 mM creatine phosphate and 10 units ml$^{-1}$ creatine phosphokinase. [$^{32}$P]cAMP was separated from [$^{32}$P]ATP by ion exchange chromatography as described previously (Salomon, 1979). Some reactions included isoproterenol, guanine nucleotides or forskolin as indicated in Results.

### *Western blots*

Equal amounts of membrane protein (40 $\mu$g) from dexamethasone-treated C10 and A5GR1 cells were separated by 10% sodium dodecyl sulphate-polyacrylamide gel electrophoresis (SDS-PAGE) as described previously (Droms *et al.*, 1989). Resolved proteins were electrophoretically transferred to BA85 nitrocellulose (Schleicher and Schuell, Keene, NH, USA) in buffer containing 190 mM glycine, 20% methanol and 25 mM Tris, pH 8.3. Nitrocellulose membranes were subsequently incubated for 1.5 h in block buffer, consisting of 5% bovine serum albumin and 0.1% NP-40 in PBS (140 mM sodium chloride, 4 mM disodium hydrogen phosphate, 3 mM potassium chloride, 1.5 mM potassium hydrogen phosphate, 0.5 mM calcium chloride, 0.5 mM magnesium chloride, pH 7.4), rinsed 3 × 10 min in PBS and incubated for 12–16 h at 4°C with gentle rocking in block buffer with either a 1 : 16 000 dilution of an anti-$G_s\alpha$ rabbit polyclonal antibody (UBI, Lake Placid, NY, USA) or with a 1 : 40 000 dilution of anti-human G$\beta$ common rabbit polyclonal antiserum (UBI). After rinsing 3 × 10 min in PBS, membranes were incubated

**Figure 1** Scatchard analysis of $^{125}$ICYP binding. Cell membranes were incubated with a range of $^{125}$ICYP concentrations. Specific binding (total $^{125}$ICYP binding minus that in the presence of 1 $\mu$M propranolol) was assayed as described in Materials and methods. A5 and A5GR1 cells cultured without dexamethasone and A5 cells cultured with 10 nM dexamethasone had few binding sites, although the exact number is uncertain as a large percentage of the total binding is non-specific and the points lie very close to the origin. In constrast, when A5GR1 cells were cultured with dexamethasone $^{125}$ICYP binding was similar to that observed in C10 cells. Each point is the mean of duplicate determinations from a representative assay. ○, C10; □, A5GR1; △, A5; open symbols, minus dexamethasone; closed symbols, + 10 nM dexamethasone.

with a 1:4000 dilution of alkaline phosphatase conjugated goat anti-rabbit secondary antibody (Cappel, Westchester, PA, USA). Detection was achieved by incubating the blots in alkaline phosphatase buffer (100 mM sodium chloride, 50 mM magnesium chloride, 100 mM Tris pH 9.5) containing 400 μM 5-bromo-4-chloro-3-indolyl-phosphate (Sigma, St Louis, MO, USA) and 400 μM nitro blue tetrazolium (Sigma). The resultant blots were scanned with a Mirror 1200 scanner (Mirror Technologies, St Paul, MN, USA) and signals were quantified using the NIH Image program.

### Results

*The number and affinity of $^{125}$ICYP binding sites are similar in dexamethasone-treated C10 and A5GR1 cells*

Some tumorigenic mouse lung-derived cell lines have reduced βAR expression compared with non-tumorigenic cells (Lange-Carter *et al.*, 1992). Both the neoplastic A5 cell line and the stable glucocorticoid receptor-transfectant cell line A5GR1 also have very few membrane-associated βAR, as determined by saturation binding of the antagonist $^{125}$ICYP (Figure 1). Culturing the A5 cell line with dexamethasone did not increase $^{125}$ICYP binding (Figure 1). However, when cultured with 10 nM dexamethasone, the A5GR1 cells exhibited similar numbers of βAR as C10 cells (Figure 1). There were no significant differences in $k_D$ or $B_{max}$ between C10 cells cultured without dexamethasone, C10 cells with 10 nM dexamethasone or A5GR1 cells with 10 nM dexamethasone [$k_D = 23 \pm 2.6$ (s.e.m.), $35 \pm 9.6$ and $30 \pm 12$ pM, $P > 0.1$; $B_{max} = 88 \pm 10$, $91 \pm 11$, $80 \pm 7.7$ fmol per mg protein, $P > 0.5$; $n = 6$, 4 and 5 respectively]. Statistical analyses were by one-way ANOVA.

**Table I** Radioimmunoassay of isoproterenol-stimulated cAMP production

| *Cell line* | *10 nM dex* | *Basal* | *1 μM iso* |
|---|---|---|---|
| A5 | – | 2.3±0.28 | 5.4±0.73 |
| | + | 2.0±0.37 | 6.2±0.47 |
| A5GR1 | – | 4.2±0.11 | 8.0±1.1 |
| | + | 2.5±0.35 | 36±7.3 |
| C10 | – | 11±1.6 | 280±38 |
| | + | 12±4.0 | 370±23 |

Intracellular cAMP production after 1 min treatment with 1 μM isoproterenol (iso) was significantly ($P < 0.01$) enhanced if C10 or A5GR1 cells were cultured for 4 days with 10 nM dexamethasone (dex), whereas A5 cell isoproterenol responsiveness was unaffected. Basal intracellular cAMP was also greater in C10 than in A5 or A5GR1 cells, but was not affected by dexamethasone treatment. Numbers represent the mean ± s.e.m. cAMP produced (pmol cAMP per mg protein). Statistical analyses were by *t*-tests. $n = 6$ for each condition.

**Table II** Basal and isoproterenol-stimulated adenylate cyclase activity

| *Cell line* | *Basal* | *100 nM iso* | *10 μM iso* |
|---|---|---|---|
| A5GR1 | 130±12 | 480±9.8 | 530±6.7 |
| C10 | 260±46 | 2700±110 | 2700±160 |

Membranes from A5GR1 cells cultured with 10 nM dexamethasone have both lower basal and lower isoproterenol (iso)-stimulated adenylate cyclase activities compared with C10 cells cultured with dexamethasone. Numbers represent the mean ± s.e.m. cAMP produced (pmol cAMP per mg protein per 20 min) from two assays ($n = 6$ for each condition).

*Dexamethasone-treated A5GR1 cells are much less isoproterenol responsive than C10 cells*

Intracellular cAMP production stimulation by the β-adrenergic agonist isoproterenol did increase approximately 5-fold when A5GR1 cells were cultured with 10 nM dexamethasone, whereas no such increase was observed in A5 cells (Table I). However, although C10 and A5GR1 cells cultured with dexamethasone had similar number of $^{125}$ICYP binding sites, A5GR1 cells remained much less responsive to isoproterenol both in whole cells (Table I) and in crude membrane fractions (Table II). The dexamethasone-induced increase in β-adrenergic responsiveness in A5GR1 cells is likely to result from the increase in βAR expression. The approximate 30% increase in isoproterenol responsiveness in C10 cells cultured with dexamethasone compared with control C10 cells was not associated with increased βAR expression and involves enhanced coupling of the βAR to $G_s$ (KA Droms, unpublished). C10 cells also exhibited a greater basal level of intracellular cAMP than did A5 or A5GR1 cells (Table I) and an increased basal adenylyl cyclase activity compared with A5GR1 cells (Table II). The increased basal adenylyl cyclase activity in C10 cells may be due to a difference in the activity of $G_s$ between cell lines (see below).

*C10 and A5GR1 cells have similar forskolin-stimulated adenylyl cyclase activities*

As both basal and isoproterenol-stimulated adenylyl cyclase activities are lower in A5GR1 than in C10 cells, a difference between cell lines in the activity of adenylyl cyclase is possible. However, when membranes prepared from C10 and A5GR1 cells cultured with dexamethasone were stimulated with 100 μM forskolin, which activates adenylyl cyclase directly (Downs and Aurbach, 1982), only a slight difference in adenylyl cyclase activity was observed (Figure 2). This difference in forskolin-stimulated adenylyl cyclase activity is much less than the isoproterenol sensitivity difference between cell lines. In fact, as the basal activity is also lower in A5GR1 cells, the fold stimulation by forskolin is greater than in C10 membranes.

**Figure 2** Forskolin-stimulated adenylyl cyclase activity. Membranes from C10 and A5GR1 cells cultured with 10 nM dexamethasone do exhibit a slight difference in forskolin-stimulated adenylyl cyclase activity, but this difference is much less than the difference between cell lines in isoproterenol sensitivity. Basal adenylyl cyclase activity is also lower in A5GR1 cells. Data from two assays are combined, $n = 6$ for each condition. □, Basal activity; ■, + 100 μM forskolin.

**Figure 3** $G_s\alpha$ and $G\beta$ Western blots. C10 (lanes 1) and A5GR1 (lanes 2) cells were cultured with 10 nM dexamethasone and membranes prepared as described in Materials and methods. Representative blots probed with anti-$G_s\alpha$ or anti-$G\beta$ are shown. No clear difference between cell lines in the amount of these subunits has been observed. Both the 45 kDa and 52 kDa molecular weight forms of $G_s\alpha$ are observed in C10 and A5GR1 cells. For comparison, S49wt type cells, which express both $G_s\alpha$ isoforms, and S49cyc⁻ cells, which do not express $G_s\alpha$ (Harris *et al.*, 1985), are also shown. The blot shown is representative of at least three blots for each subunit.

### *C10 and A5GR1 cells have similar levels of $G_s\alpha$ and $G\beta$ subunit expression*

In some cases, the relative levels of expression of G protein subunits can regulate receptor–G protein coupling (Blumer and Thorner, 1990). Therefore, $G_s\alpha$ and $G\beta$ subunit expression were examined on Western immunoblots (Figure 3). For each subunit, three individual blots were quantified as indicated in Materials and methods and the signal from C10 cells was set to 1 (signals from the 45 kDa and 52 kDa forms of $G_s\alpha$ were combined). A5GR1 cells did not differ significantly ($P>0.5$ for both subunits) from C10 for either $G_s\alpha$ (1.1±0.31 s.e.m.) or $G\beta$ (0.91±0.33) expression. Thus, no clear differences between cell lines were observed in the amounts of any of the components of the βAR-coupled adenylyl cyclase system that might explain their differences in hormone responsiveness.

### *The βAR and $G_s$ are functionally uncoupled in A5GR1 cells*

When A5GR1 and C10 cells are cultured with dexamethasone they express similar amounts of βAR and $G_s$ and have similar forskolin-stimulated adenylyl cyclase activities, yet the A5GR1 cells are much less responsive to isoproterenol. Therefore, the interactions between the components of the βAR-coupled adenylyl cyclase system were examined. βAR–$G_s$ coupling was analysed by isoproterenol competition for

**Figure 4** Isoproterenol competition for $^{125}$ICYP binding. Both C10 (**a**) and A5GR1 (**b**) cell membranes exhibited low- and high-affinity isoproterenol binding when incubated without guanine nucleotide. When 8 μM GTP was included, C10 had only low-affinity isoproterenol binding sites. In constrast, even 40 μM GTP only minimally reduced isoproterenol affinity in A5GR1 membranes. Membranes were prepared from cells cultured with 10 nM dexamethasone and incubated with 30 pM $^{125}$ICYP and the indicated concentrations of unlabelled isoproterenol. Data were analysed using the Ligand program (Munson and Rodbard, 1983). Each point is the mean of duplicate determinations from one assay, whereas each line is the best fit from Ligand analysis of the combined data from at least three independent assays, except for A5GR1 -GTP for which two assays were performed (see Table I). Open symbols, minus GTP; closed symbols, +GTP (8 μM for C10, 40 μM for A5GR1).

**Table III** Summary of competition binding data

| *Cell line* | *[GTP]* | $k_H$ *(nM)* | $k_L$ *(nM)* | $\%R_H$ | n |
|---|---|---|---|---|---|
| C10 | – | 14±2.6 | 1500±430 | 68±4.1 | 4 |
| | 8 μM | – | 510±72 | 0 | 3 |
| A5GR1 | – | 12±2.0 | 230±20 | 60±1.5 | 2 |
| | 40 μM | 30±16 | 330±70 | 48±10 | 3 |

Membranes from cells cultured with 10 nM dexamethasone were incubated with $^{125}$ICYP, varying isoproterenol concentrations and the GTP concentrations indicated. The $k_D$s for high ($k_H$) and low ($k_L$) affinity isoproterenol binding and the percentage of high-affinity sites ($\%R_H$) are reported. Numbers are the mean±s.e.m. of parameters estimated from Ligand analysis of individual assays. The number of individual assays for each condition is also reported (n).

$^{125}$ICYP binding in the presence or absence of GTP. In such assays, $\beta$-adrenergic receptors exhibit two affinity states for agonist binding: one of higher affinity ($k_H$) and the other of lower affinity ($k_L$). As demonstrated in Figure 4 and Table III, both C10 and A5GR1 cells exhibit high- and low-affinity isproterenol binding in the absence of GTP. The presence of high-affinity sites in both cell lines indicates that there is $\beta$AR–$G_s$ 'coupling' in both cell lines (De Lean *et al.*, 1980). However, inclusion of GTP in the incubations is much more effective at reducing high-affinity isoproterenol binding in C10 than in A5GR1 cells, indicating greater functional $\beta$AR–$G_s$ coupling in the former (Rodbell *et al.*, 1971; Cheung *et al.*, 1989). It is also interesting to note that $k_L$ is much greater in C10 than in A5GR1 cells in the absence of GTP (Table III). This high $k_L$ is typically observed when $\beta$AR–$G_s$ coupling is enhanced by glucocorticoid treatment (Davies and Lefkowitz, 1981), and is consistent with the increased isoproterenol responsiveness that was observed in C10 cells cultured with dexamethasone (Table I). The $k_L$ for C10 cells (140±22 nM) that were not cultured with dexamethasone is similar to that for A5GR1 cells (230±20 nM).

**Figure 5** Guanine nucleotide stimulation of adenylye cyclase. (**a**) GTP produced a concentration-dependent activation of adenylyl cyclase in membranes prepared from C10 cells that were cultured with dexamethasone, but was completely ineffective in A5GR1 cells cultured with dexamethasone. (**b**) The non-hydrolysable analogue Gpp(NH)p activated adenylyl cyclase in both cell lines, but to a lesser extent in A5GR1 than in C10 cells. Stimulation of adenylyl cyclase by Gpp(NH)p in A5GR1 cells that were not cultured with dexamethasone and, thus expressed very few $\beta$AR, was the same as for A5GR1 cells cultured with dexamethasone. Points represent the combined data from three independent assays for which triplicate determinations were done, with the exception of A5GR1 without dexamethasone, for which one assay was performed. ○, C10; ■, A5GR1; open symbols, minus dexamethasone; closed symbols, +10 nM dexamethasone.

*Guanine nucleotides stimulate adenylyl cyclase more effectively in C10 than in A5GR1 cells*

Neither GTP nor the non-hydrolysable analogue 5-guanylyimidio-diphosphate [Gpp(NH)p] activated adenylyl cyclase as effectively in A5GR1 cell membranes as in C10 membranes (Figure 5). In fact, GTP at concentrations as high as 100 $\mu$M produced no stimulation of adenylyl cyclase above basal in A5GR1 cells. Although Gpp(NH)p was also much less effective in A5GT1 than in C10 cells, 15 $\mu$M Gpp(NH)p did stimulate adenylyl cyclase approximately 3-fold above basal activity in the former. As adenylyl cyclase was stimulated by Gpp(NH)p to a similar extent in A5GR1 cells that either were or were not cultured with dexamethasone (Figure 5), a defect in $G_s$ that is independent of $\beta$AR expression is implicated.

### Discussion

Mouse lung tumour cells have considerably reduced sensitivity to $\beta$-adrenergic stimulation (Droms *et al.*, 1989; Lange-Carter *et al.*, 1992) and this loss of sensitivity involves reduced $\beta$AR expression (Lange-Carter *et al.*, 1992). The present work indicates that glucocorticoid treatment of A5GR1 cells, a tumour cell line stably expressing a glucocorticoid receptor transgene, does allow restoration of $\beta$AR expression to a level similar to that observed in the non-tumorigenic C10 cells. The newly expressed $\beta$AR is capable of high-affinity ternary complex formation with agonist and $G_s$, as indicated by the observation that 60% of isoproterenol binding sites are high affinity (Table III). Reduced tumour cell $\beta$AR expression is unlikely to result directly from loss of glucocorticoid stimulation, as C10 cells retain a high level of $\beta$AR expression even in the absence of glucocorticoids in the culture medium. Thus, although glucocorticoid treatment does restore $\beta$AR expression in A5GR1 cells, the cause of reduced expression in tumour cells is unknown.

Mouse lung tumour cells also exhibited reduced guanine nucleotide analogue binding to a 45 kDa membrane-associated protein and enhanced cholera toxin responsiveness (Droms *et al.*, 1989; Lange-Carter *et al.*, 1992), suggesting the hypothesis that G protein function is altered in the mouse lung tumours. A functional alteration in tumour cell $G_s$ could result from the reduced $\beta$AR expression rather than from a direct defect in $G_s$ itself, as even unoccupied $\beta$AR can influence G protein activity (Bond *et al.*, 1995). However, the present study indicates that functional $\beta$AR–$G_s$ coupling is considerably reduced in A5GR1 cells even when these cells express as many $\beta$AR as C10 cells. This lack of functional $\beta$AR–$G_s$ coupling in A5GR1 cells is indicated by a relative inability of GTP to destablise the high-affinity ternary complex and is associated with decreased effectiveness of guanine nucleotides to activate adenylyl cyclase. As A5GR1 cells exhibit a decrease in non-hydrolysable guanine nucleotide activation of adenylyl cyclase that is independent of glucocorticoid treatment and, consequently, $\beta$AR expression, an independent defect in tumour cell $G_s$ is implicated.

One potential mechanism of the reduced ability of guanine nucleotides to activate $G_s$ in tumorigenic mouse lung-derived cell lines is that guanine nucleotide exchange is reduced (Droms *et al.*, 1989). The relative inability of GTP to destabalise the ternary complex in A5GR1 cells is consistent with this hypothesis. Additionally, the difference between C10 and A5GR1 cells in the effectiveness of GTP at activating adenylyl cyclase is even greater than the difference between cell lines when a non-hydrolysable analogue is used. Thus, the GTPase activity of $G_s$ may also be enhanced in the tumour cells. A reduction in the intrinsic guanine nucleotide exchange rate with an increase in the GTPase activity of $G_s$ in tumour cells would be predicted to produce a decrease in basal adenylyl cyclase activity, consistent with the 2- to 3-fold lower basal intracellular cAMP and adenylyl cyclase activity observed in A5 and A5GR1 cells compared with C10 cells.

Although a clear role for reduced intracellular cAMP in neoplasia has not been established, agonist-stimulated adenylyl cyclase activity is reduced in many tumours. One possibility is that cAMP interferes with mitogenic signal transduction, as observed in Rat1 cells (Cook and McCormick, 1993) and human small-cell lung cancer cells (Viallet *et al.*, 1990). Reduced activity of the cAMP-dependent protein kinase is also a requirement for mitogenesis in some cells (Lamb *et al.*, 1991). In addition, cAMP inhibits growth in soft agar of mouse lung tumour cell lines (KA Droms, unpublished observation). Tumour-associated reductions in cAMP often result from decreases in the number or affinity of receptors coupled to adenylyl cyclase activation (Hunt and Martin, 1980). Alternatively, defects in the G-proteins that couple receptors to adenylyl cyclase may also occur. For example, reduced GTP-dependent coupling of adrenergic receptors to adenylyl cyclase had been observed in hepatomas (Okamura and Terayama, 1976). As there are differences between C10 and A5GR1 cells in $G_s$ interactions with both the $\beta$AR (guanine nucleotide destabilisation of the ternary complex) and adenylyl cyclase (guanine nucleotide activation of cAMP production), the structure of one or more of the subunits of $G_s$ may be altered in mouse lung tumour cells. This possibility is currently being investigated.

**Abbreviations**

$\beta$AR, $\beta$-adrenergic receptor; FBS, fetal bovine serum; $G_s$, stimulatory heterotrimeric guanine nucleotide binding protein; Gpp(NH)p, 5′-guanylylimidodiphosphate; $^{125}$ICYP, $(-)^{125}$I-cyanopindolol; PBS, phosphate-buffered saline; SDS-PAGE, sodium dodecyl sulphate polyacrylamide gel eletrophoresis; TCA, trichloroacetic acid.

**Acknowledgements**

The author thanks Jerry Allen, Kenneth Chen and Erin Eisen for technical assistance and Dr Laurel M Donahue for helpful comments on the manuscript. This work was supported by grants from the Texas Tech University School of Medicine Seed Research Grant Program and the American Heart Association, Texas Affiliate grant no. 93G-330.

### References

BALLARD PL. (1986). Hormones and lung maturation. *Monographs Endocrinol.*, **28,** 24–193.

BARNES PJ, JACOBS MM AND ROBERTS JM. (1984). Glucocorticoids preferentially increase fetal alveolar $\beta$-adrenoreceptors: autoradiographic evidence. *Pediatr. Res.*, **18,** 1191–1194.

BENTEL JM, LYKKE AWJ AND SMITH GJ. (1989). Cloned murine non-malignant, spontaneously transformed and chemical tumour-derived cell lines related to the type 2 pneumocyte. *Cell Biol. Int. Rep.*, **13,** 729–738.

BLUMER KJ AND THORNER J. (1990). $\beta$ and $\gamma$ subunits of a yeast guanine nucleotide-binding protein are not essential for membrane association of the $\alpha$ subunit but are required for receptor coupling. *Proc. Natl Acad. Sci. USA*, **87,** 4363–4367.

BOND RA, LEFF P, JOHNSON TD, MILANO CA, ROCKMAN HA, MCMINN TR, APPARSUNDARAM S, HYEK MF, KENAKIN TP, ALLEN LF AND LEFKOWITZ RJ. (1995). Physiological effects of inverse agonists in transgenic mice with myocardial overexpression of the $\beta_2$-adrenoceptor. *Nature*, **374,** 272–276.

CHENG JB, GOLDFIEN A, BALLARD PL AND ROBERTS JM. (1980). Glucocorticoids increase pulmonary $\beta$-adrenergic receptors in fetal rabbit. *Endocrinology*, **107,** 1646–1648.

CHEUNG AH, SIGAL IS, DIXON RAF AND STRADER CD. (1989). Agonist-promoted sequestration of the $\beta_2$-adrenergic receptor requires regions involved in functional coupling with $G_s$. *Mol. Pharmacol.*, **35,** 132–138.

COLLINS S, CARON MG AND LEFKOWITZ RJ. (1988). $\beta_2$-adrenergic receptors in hamster smooth muscle cells are transcriptionally regulated by glucocorticoids. *J. Biol. Chem.*, **263,** 9067–9070.

COOK PW, SWANSON KT, EDWARDS CP AND FIRESTONE GL. (1988). Glucocorticoid receptor-dependent inhibition of cellular proliferation in dexamethasone-resistant and hypersensitive rat hepatoma cell variants. *Mol. Cell. Biol.*, **8,** 1449–1459.

COOK SJ AND MCCORMICK F. (1993). Inhibition by cAMP of Ras-dependent activation of Raf. *Science*, **262,** 1069–1072.

DAVIES AO AND LEFKOWITZ RJ. (1981). Agonist-promoted high affinity state of the $\beta$-adrenergic receptor in human neutrophils: modulation by corticosteroids. *J. Clin. Endocrinol. Metab.*, **53,** 703–708.

DAVIES AO AND LEFKOWITZ RJ. (1984). Regulation of $\beta$-adrenergic receptors by steroid hormones. *Annu. Rev. Physiol.*, **46,** 119–130.

DE LEAN A, STADEL JM AND LEFKOWITZ RJ. (1980). A ternary complex model explains the agonist-specific binding properties of the adenylyl cyclase-coupled $\beta$-adenergic receptor. *J. Biol. Chem.*, **255,** 7108–7117.

DOWNS JRW AND AURBACH GD. (1982). The effect of forskolin on adenylyl cyclase in S49-wild type and CYC(–)-cells. *J. Cyclic Nucleotide Res.*, **8,** 235–242.

DROMS KA. (1995). Dexamethasone enhances colonization of soft agar by tumorigenic mouse lung-derived cell lines. *Cancer Lett.*, **95,** 99–103.

DROMS KA, HALEY BE, SMITH GJ AND MALKINSON AM. (1989). Decreased $8N_3$-[$\gamma$-$^{32}$P]GTP photolabeling of $G_s\alpha$ in tumorigenic lung epithelial cell lines: association with decreased hormone responsiveness and loss of contact-inhibited growth. *Exp. Cell Res.*, **182,** 330–339.

DROMS KA, HANSON LA, MALKINSON AM AND BEER DG. (1993). Altered dexamethasone responsiveness and loss of growth control in tumorigenic mouse lung cell lines. *Int. J. Cancer.*, **53,** 1017–1022.

HARRIS BA, ROBISHAW JD, MUMBY SM AND GILMAN AG. (1985). Molecular cloning of complementary DNA for the alpha subunit of the G protein that stimulates adenylyl cyclase. *Science*, **229,** 1274–1277.

HUNT NH AND MARTIN TJ. (1980). Hormone receptors and cyclic nucleotides; significance for growth and function of tumors. *Mol. Aspects Med.*, **3,** 59–118.

JOHNSON GS AND JAWORSKI CJ. (1983). Glucocorticoids increase GTP-dependent adenylyl cyclase activity in cultured fibroblasts. *Mol. Pharmacol.*, **23,** 648–652.

LAMB NJ, CAVADORE JC, LABBE JC, MAURER RA AND FERNANDEZ A. (1991). Inhibition of cAMP-dependent protein kinase plays a key role in the induction of mitosis and nulcear envelope breakdown in mammalian cells. *EMBO J.*, **10,** 1523–1533.

LANGE-CARTER CA, DROMS KA, VUILLEQUEZ JJ AND MALKINSON AM. (1992). Differential responsiveness to agents which stimulate cAMP production in normal versus neoplastic mouse lung epithelial cells. *Cancer Lett.*, **67,** 139–144.

LEVITZKI A. (1988). From epinephrine to cyclic AMP. *Science*, **241,** 800–806.

LOWRY OH, ROSEBROUGH NJ, FARR AL AND RANDALL RJ. (1951). Protein measurement with the Folin phenol reagent. *J. Biol. Chem.*, **193,** 265–275.

MUNSON PJ AND RODBARD D. (1983) LIGAND: a versatile computerized approach for characterization of ligand binding systems. *Anal. Biochem.*, **107,** 220–239.

OKAMURA N AND TERAYAMA H. (1976). Comparison of the epinephrine-mediated activation of adenylyl cyclase in plasma membranes from liver and ascites hepatomas of rats. *Biochim. Biophys. Acta*, **455,** 297–314.

RODBELL M, KRANS MJ, POHL SL AND BIRNBAUMER L. (1971). The glucagon-sensitive adenyl cyclase system in plasma membranes of rat liver. IV. Effects of guanyl nucleotides on binding of $^{125}$I-glucagon. *J. Biol. Chem.*, **246,** 1872–1876.

SALOMON Y. (1979). adenylyl cyclase assay. *Adv. Cyclic Nucleotide Res.*, **10,** 35–54.

VALVERIUS P, HOFFMAN PL AND TABOKOFF B. (1987). Effect of ethanol on mouse cerebral cortical beta-adrenergic receptors. *Mol. Pharmacol.*, **32,** 217–222.

VIALLET J, SHARONI Y, FRUCHT H, JENSEN RT, MINNA JD AND SAUSVILLE EA. (1990). Cholera toxin inhibits signal transduction by several mitogens and the in vitro growth of human small-cell lung cancer. *J. Clin. Invest.*, **86,** 1904–1912.